# YAP promotes global mRNA translation to fuel oncogenic growth despite starvation

**DOI:** 10.1038/s12276-024-01316-w

**Published:** 2024-10-01

**Authors:** Daehee Hwang, Seonguk Baek, Jeeyoon Chang, Taejun Seol, Bomin Ku, Hongseok Ha, Hyeonji Lee, Suhyeon Cho, Tae-Young Roh, Yoon Ki Kim, Dae-Sik Lim

**Affiliations:** 1 National Creative Research Initiatives Center for Cell Plasticity, KAIST Stem Cell Center, Department of Biological Sciences, Korea Advanced Institute of Science and Technology (KAIST), Daejeon, 34141 Republic of Korea; 2 Department of Biological Sciences, Korea Advanced Institute of Science and Technology (KAIST), Daejeon, 34141 Republic of Korea; 3 Transdisciplinary Department of Medicine and Advanced Technology, Seoul National University Hospital, Seoul, 03080 Republic of Korea; 4 Department of Life Sciences, Pohang University of Science and Technology (POSTECH), Pohang, 37673 Republic of Korea; 5 Department of Life Sciences, Ewha Womans University, Seoul, 03760 Republic of Korea

**Keywords:** Translation, Cell signalling, Functional genomics, Cancer genetics

## Abstract

Yes-associated protein (YAP) and transcriptional co-activator with PDZ-binding motif (TAZ) play fundamental roles in stem/progenitor cell expansion during homeostasis, and their dysregulation often leads to tissue overgrowth. Here, we show that YAP activation is sufficient to overcome the restriction of global protein synthesis induced by serum starvation, enabling cells to sustain proliferation and survival despite an unfavorable environment. Mechanistically, YAP/TAZ selectively promoted the mTORC1-dependent translation of mRNAs containing 5′ terminal oligopyrimidine (5′TOP) motifs, ultimately increasing the cellular polysome content. Interestingly, DNA damage-inducible transcript 4 (DDIT4), a negative regulator of mTORC1, was upregulated by serum starvation but repressed by YAP/TAZ. DDIT4 was sufficient to suppress the translation and transformative potential of uveal melanoma cells, which are often serum unresponsive due to G protein mutations. Our findings reveal a vital role for protein synthesis as a key modality of YAP/TAZ-induced oncogenic transformation and indicate the potential for targeting mTORC1 or translation to treat YAP/TAZ-driven malignancies.

## Targeting the translation machinery offers hope for YAP/TAZ-driven cancers

This research investigates how cells manage their size and proliferation by coordinating two signaling pathways, Hippo and mTOR. As these pathways are fundamental for normal development, their dysregulation results in numerous diseases, including many cancers. In particular, the study aims to understand how YAP and TAZ—effectors of the Hippo pathway—influence mTOR-mediated protein synthesis in cells, a previously unclear process. Surprisingly, our findings show that YAP and TAZ can maintain active protein synthesis even when cells are deprived of nutrients in both cultured cells and mice. Since self-sufficiency in growth signals is a key hallmark of cancer, targeting this axis could serve as a novel and effective therapeutic strategy. This summary was initially drafted using artificial intelligence, then revised and fact-checked by the author.

## Introduction

Yes-associated protein (YAP) and its paralog transcriptional co-activator with PDZ-binding motif (TAZ) are potent transcriptional coactivators of the evolutionarily conserved Hippo tumor suppressor pathway^1^. In mammals, a kinase cascade initiated by MST1/2 (mammalian sterile 20-like kinase) phosphorylates LATS1/2 (large tumor suppressor kinase)^2^. Activated LATS1/2 then phosphorylates YAP/TAZ, promoting their cytoplasmic sequestration and proteolytic degradation^3^. On the other hand, dephosphorylated YAP/TAZ readily translocate to the nucleus, where these proteins bind to the TEAD family of transcription factors to upregulate genes involved in progenitor cell expansion and tissue homeostasis^4,5^ or downregulate genes involved in apoptotic or tumor suppressive roles^6,7^. Consequently, unrestrained induction of YAP/TAZ target genes culminates in diverse cancer hallmark traits, including driving cell cycle progression^8,9^, evading cell death^10^, inducing angiogenesis^11,12^, promoting invasion and metastasis^13^, and enabling growth under adverse conditions^14,15^. However, studies to date have focused only on direct YAP/TAZ targets or distal chromatin interactions at the transcriptional level^16^, limiting our knowledge as to whether YAP/TAZ contributes to the cellular translatome.

Polypeptide products ultimately perform the functions encoded within the mRNA. Moreover, rRNA and tRNA, which are required for the translation of these mRNAs, together constitute more than 90% of total cellular RNA by mass^17^. Therefore, understanding the biomolecular mechanisms that affect mRNA translation and the physiological effects that ensue is essential. In particular, how mRNA translation is affected by oncogenic signals and contributes to cancer propagation is not fully understood. mRNA translation is regulated primarily by mammalian target of rapamycin complex 1 (mTORC1), with a particular emphasis on transcripts that bear a 5′ terminal oligopyrimidine (5′TOP) tract immediately downstream of the 7-methylguanosine triphosphate (m^7^GTP) cap^18^. These 5′TOP-containing mRNAs encode many components of the translational machinery, such as ribosomal proteins and translation factors^19^. mTORC1 responds to diverse extracellular cues, including nutrients, growth factors, energy, and stress^20^. Activated mTORC1 phosphorylates many substrates, among which p70 ribosomal protein S6 kinase 1 (S6K1), eukaryotic initiation factor 4E binding proteins (4E-BP), and Unc51-like autophagy activating kinase 1 (ULK1) are the most well defined. S6K1-mediated phosphorylation of ribosomal protein S6 promotes ribosome recruitment to the preinitiation complex at the 5′ end of mRNA transcripts^21^. Likewise, 4E-BP phosphorylation prevents its interaction with eIF4E, the latter of which becomes free to join other members of the translation initiation complex, such as eIF4G and eIF4A^22^.

Given that the Hippo–YAP–TAZ and mTORC1 signaling pathways are critical for regulating organ size by controlling cell number and size, respectively, research into their mechanistic crosstalk remains active and progressive. An earlier study reported that YAP upregulates miR-29 to downregulate PTEN, a negative regulator of the PI3K-AKT-mTORC1 pathway^23^. A recent study reported another mechanism in which LATS directly phosphorylates Raptor, an essential component of mTORC1, leading to reduced binding to the mTORC1 activator Rheb^24^. Indirect mechanisms by which YAP activates mTORC1 have also been defined, for example, by inducing the expression of amino acid transporters, which consequently activate mTORC1^25,26^. Previously, we reported that YAP-TEAD transcriptionally represses the expression of DNA damage-inducible transcript 4 (*DDIT4*; also known as *REDD1*)^6^. DDIT4 is an established inhibitor of mTORC1 that acts to increase the functional pool of TSC1/2, a direct upstream regulator of mTORC1 that represses Rheb by virtue of its GTPase activity^27^. Interestingly, *DDIT4* expression is induced by various stress signals not limited to DNA damage, such as nutrient deprivation^28^, hypoxia^29^, and heat shock^30^. However, whether YAP/TAZ ultimately promotes mRNA translation, a defining feature of mTORC1 activation and the most energy-demanding process in the cell^31^, has never been studied in detail. Moreover, if so, under which physiological context this effect becomes most prominent also remains unclear.

In cultured cells, the expression of active YAP positively affects mTORC1 activity and increases general translation dynamics. This effect becomes evident when cells are starved of serum, which, under normal circumstances, terminates translation^32^. At the molecular level, *DDIT4* expression is induced by serum starvation, and YAP overexpression blocks this effect. Notably, DDIT4 expression is sufficient to block YAP-mediated translation under serum-starved conditions. Physiologically, we demonstrate that DDIT4 overexpression or pharmacological inhibition of mTORC1 effectively abrogates the oncogenic potential of serum-unresponsive uveal melanoma cells, which commonly originate from mutations in G protein-coupled receptor (GPCR)-encoding genes^33,34^. Collectively, our findings advocate the use of inhibitors against mTORC1 or mRNA translation for therapeutic targeting of YAP/TAZ-driven malignancies.

## Materials and methods

### Antibodies

Antibodies against the following antigens were used for immunoblot analysis: puromycin (Kerafast, EQ0001), FLAG (Sigma-Aldrich, F3165), YAP/TAZ (Cell Signaling Technology, #8418), YAP (Cell Signaling Technology, #14074), TEAD4 (Abcam, ab58310), CYR61 (Santa Cruz Biotechnology, sc-13100), DDIT4 (Novus Biologicals, NBP1-22966), mTOR (Cell Signaling Technology, #2972), phospho-S6 (Cell Signaling Technology, #2211), S6 (Cell Signaling Technology, #2217), phospho-4E-BP1 (Cell Signaling Technology, #2855), 4E-BP1 (Cell Signaling Technology, #9644), phospho-ERK (Cell Signaling Technology, #9101), ERK1/2 (Cell Signaling Technology, #4695), phospho-S6K1 (Cell Signaling Technology, #9205), S6K1 (Cell Signaling Technology, #9202), phospho-AKT (Cell Signaling Technology, #4060 and #4056), AKT2 (Cell Signaling Technology, #3063), eIF4E (Santa Cruz Biotechnology, sc-9976), PABP-C1 (Abcam, ab21060), phospho-paxillin (Cell Signaling Technology, #2541), phospho-p38 (Cell Signaling Technology, #4511), and vinculin (Cell Signaling Technology, #13901).

### Animals

*Lats1*^*fl/fl*^; *Lats2*^*fl/fl*^;LSL-tdTomato mice were generated as described previously^26^. Conditional deletion of *Lats1/2* in the livers of male mice at 12 weeks of age was induced via intraperitoneal injection of 5 × 10^11^ gc of AAV8-TBG-Cre (Addgene #107787-AAV8) or AAV8-TBG-GFP to serve as a control (Addgene #105535-AAV8). The animals were randomly assigned to treatment groups. The investigators were not blinded to the treatment. All animal care and experiments were performed under guidelines approved by the Biomedical Research Center of KAIST.

### Cell lines and cell culture

MCF10A cells were cultured in Dulbecco’s modified Eagle’s medium (DMEM)-F12 (Welgene) supplemented with 5% horse serum (Invitrogen), epidermal growth factor (EGF, 20 ng/ml; Peprotech), insulin (10 μg/ml, Sigma), hydrocortisone (0.5 μg/ml, Sigma), cholera toxin (100 ng/ml, Sigma), and penicillin‒streptomycin (Invitrogen). AML12 cells were cultured in DMEM-F12 supplemented with 10% fetal bovine serum (FBS, Invitrogen), insulin–transferrin–selenium (1%, Invitrogen), dexamethasone (40 ng/ml, Sigma), and penicillin‒streptomycin. In addition, 293 T, 293AD and HaCaT cells were cultured in DMEM (Welgene) supplemented with 10% FBS and penicillin‒streptomycin. RPE-1 cells were cultured in DMEM-F12 supplemented with 10% FBS and penicillin‒streptomycin, and 92.1 and OCM1 cells were cultured in RPMI-1640 (Welgene) supplemented with 10% FBS and penicillin‒streptomycin. The cells were subjected to serum starvation in the corresponding growth medium lacking serum, with the exception of MCF10A cells, for which both serum and EGF were removed. Unless otherwise indicated, serum starvation was performed overnight for 14 h. “Serum-replete” refers to cells that have not undergone serum starvation, whereas “Serum readdition” refers to serum stimulation of serum-starved cells for 1 h prior to analysis. All cell lines were obtained from the American Type Culture Collection, with the exception of 293AD, which was provided by Kyoung-Jin Oh (KRIBB, Daejeon, Korea), and 92.1 and OCM1, which were provided by Joon Kim (KAIST, Daejeon, Korea). The cell lines were validated using DNA fingerprinting of the *TPOX*, *TH01*, *vWA*, and *D5S818* loci. The cells were routinely tested for the presence of mycoplasma by staining with 4′,6′-diamidino-2-phenylindole.

### Plasmid constructs and virus generation

Stable expression of FLAG-tagged YAP 5SA or YAP 5SA-S94A mutant forms of human YAP in cell lines was achieved by using the pMSCV hygro (Clontech) vector. Retroviral particles were prepared by transfecting 293T cells with the YAP vector together with vectors for gag/pol (Addgene #14887) and VSV.G (Addgene #14888) at a ratio of 3:2:1 using polyethylenimine (PEI). Fresh media was replaced 12 h after transfection to mitigate toxicity. At 48 h, the media containing the viral particles were collected and centrifuged at 150 × *g* for 20 min to remove the cellular debris. This virus-containing supernatant was then applied to infect target cells using polybrene (6 μg/ml, Sigma). After 24 h, the cells were subjected to selection with hygromycin (250 μg/ml, Invitrogen) for 48 h. To generate stable cell lines harboring a doxycycline-inducible DDIT4 system, the open reading frame for human DDIT4 was cloned and inserted into the pRetroX-Tight-Hyg (Clontech) vector. Given that pRetroX is a two-vector system, we first generated stable cell lines expressing pRetroX-Tet-On Advanced (Clontech) that had been selected with G418 (3 μg/ml, Sigma) for 2 weeks.

### RNA interference

Custom siRNA sequences were synthesized by GenePharma. The cells were transfected with siRNAs (20 nM) via Lipofectamine RNAiMAX (Invitrogen) and analyzed after 48 h. The sequences of the siRNAs are listed below.

**Table:** 

Gene	Target sequence (5’-3’)
Control (Con)	CGU ACG CGG AAU ACU UCG A
Human YAP	GAC AUC UUC UGG UCA GAG A
Human TAZ	ACG UUG ACU UAG GAA CUU U
Human TEAD1/3/4	GAU CAA CUU CAU CCA CAA GCU

### RNA isolation and qRT‒PCR analysis

Total RNA was isolated from cells using the RiboEx reagent (GeneAll) according to the manufacturer’s instructions. RNA (2 μg) was incubated at 70 °C for 5 min before RT with M-MLV reverse transcriptase (Enzynomics) and a 1:1 mixture of random hexamer and oligo(dT) (dT_18_) primers for 2 h at 37 °C. The resulting cDNAs were diluted with an equal volume of water and subjected to real-time qPCR analysis using a Bio-Rad CFX Connect machine. The abundance of target mRNAs was normalized to that of 18S (human) or 18S (mouse) rRNA. The sequences of the qPCR primers used are listed below.

**Table:** 

Gene	Forward sequence (5’-3’)	Reverse sequence (5’-3’)
Human 18S	CAA TTA CAG GGC CTC GAA AG	AAA CGG CTA CCA CAT CCA AG
Human CYR61	CCT TGT GGA CAG CCA GTG TA	ACT TGG GCC GGT ATT TCT TC
Human DDIT4	TGG GCA AAG AAC TAC TGC G	AGA GTT GGC GGA GCT AAA CAG
Mouse 18s	GCA ATT ATT CCC CAT GAA CG	GGC CTC ACT AAA CCA TCC AA
Mouse Cyr61	CTG CGC TAA ACA ACT CAA CGA	GCA GAT CCC TTT CAG AGC GG
Mouse Ddit4	TAC TGC CCA CCT TTC AGT TG	GTC AGG GAC TGG CTG TAA CC

### SUnSET (surface sensing of translation) assay

The cells were treated with puromycin (10 μg/ml, Invitrogen) for 10 min at 37 °C immediately before analysis. The samples were then scraped in ice-cold phosphate-buffered saline (PBS) and lysed in ice-cold radioimmunoprecipitation (RIPA) buffer, after which the lysates were precleared by centrifugation at 15,000 × *g* for 20 min at 4 °C. The resulting lysates (20 μg) were subjected to SDS‒polyacrylamide gel electrophoresis followed by immunoblot analysis with an antibody against puromycin (Kerafast, EQ0001). The blot membrane was briefly stained with Fast Green FCF (Sigma) and imaged after protein transfer prior to blocking nonspecific sites with skim milk (5%, LPS solutions). All experiments were performed with at least three biological (independent) replications, and representative results are shown.

### Phos-tag gel electrophoresis

Phos-tag gels were used to assess the YAP phosphorylation status. Briefly, Phos-tag reagent (Wako chemicals, #AAL-107) and MnCl_2_ were added to the separating gel mixture (7.5%) at final concentrations of 62.5 μM and 0.2 mM, respectively. The cell lysates were loaded onto Phos-tag gels, which were run at low voltage (60 V) until the bromophenol blue dye reached the separating gel, at which point the voltage was increased (110 V) until the 50 kDa molecular weight marker (Intron Bio) reached the bottom of the gel. The proteins were transferred to PVDF membranes (Millipore) at 400 mA for 100 min on ice. Subsequent blocking, antibody treatment, and immunoblotting steps were performed as usual.

### Polysome fractionation

Polysome fractionation was performed using 293AD cells stably expressing the empty vector or F-YAP 5SA, which were serum starved overnight. Following treatment with cycloheximide (100 μg/ml, Sigma) for 10 min, the cells were collected in ice-cold PBS containing cycloheximide (100 μg/ml, Sigma) and lysed with polysome lysis buffer [MOPS (50 mM, Sigma), MgCl_2_ (15 mM, Sigma), NaCl (150 mM, Duchefa), cycloheximide (100 μg/ml, Sigma), Triton-X-100 (0.5% (v/v), heparin sodium salt (1 mg/ml, Sigma), RiboLock RNase inhibitor (0.2 U/μl, Thermo Fisher), PMSF (2 mM, Sigma), and benzamidine hydrochloride (1 mM, Sigma)]. Then, the cell lysates were placed onto a 10–50% sucrose gradient bar and centrifuged for 2 h at 36,000 rpm at 4 °C. Fractions were collected using a Foxy Jr density gradient system (Teledyne ISCO) in accordance with A254.

### Ribo-seq library construction

Briefly, 293AD cells stably expressing the empty vector or YAP 5SA and subjected to serum starvation were preincubated with cycloheximide (100 μg/ml, Sigma) for 10 min. The cells were then lysed in 600 μl of lysis buffer [Tris-Cl pH 7.4 (10 mM, Duchefa), MgCl_2_ (5 mM, Junsei), KCl (100 mM, Junsei), Triton-X-100 (1%, Sigma), cycloheximide (100 μg/ml, Sigma), dithiothreitol (1 mM, Sigma), RiboLock RNase inhibitor (0.2 U/μl, Thermo Fisher), and EDTA-free protease inhibitor cocktail (1x, Roche)]. Then, 300 μl of the cell lysates were treated with RNase I (5 U/μl, Ambion) for 45 min at room temperature for Ribo-seq library construction. The remaining 300 μl of cell lysate was aliquoted for mRNA-seq library construction. RNase I-treated cell lysates were then placed in Illustra MicroSpin S-400 HR columns (GE Healthcare) and centrifuged at 600 × *g* for 2 min. Next, the RiboMinus TM Eukaryote System v2 (Thermo Fisher) was used for the removal of ribosomal RNAs. For processing the 5′- and 3′-ends of ribosome-protected fragments (RFPs) for subsequent library construction, Antarctic phosphatase (NEB) and T4 polynucleotide kinase (NEB) were used, and then size selection of 26–32 nt RPFs was performed using a 12% TBE-urea gel system (National Diagnostics). The end-processed and size-selected RPFs were then subjected to a TruSeq Small RNA Library Preparation Kit (Illumina) for cDNA library construction. Sequencing was performed with the Hi-seq 2500 platform (Illumina).

### Ribo-seq analysis

The adapter sequences of the Ribo-seq and mRNA-seq data were removed with Cutadapt version 3.7 (Ribo-seq; -q 30 -m 15 -a TGGAATTCTCGGGTGCCAAGG/mRNA-seq; -q 30 -m 15 -a AGATCGGAAGAGC). The contaminated rRNA sequences of the Ribo-seq data were then depleted using ribopicker (version 0.4.3). The remaining reads from the Ribo-seq data and the adapter trimmed mRNA-seq data were subsequently mapped to the genome (hg19 version) using STAR 2.5.2b (--seedSearchStartLmax 15 --outFilterMatchNmin 15 --outSAMstrandField intronMotif --outSAMattributes NH HI NM MD AS XS --outFilterMultimapNmax 5 --outFilterMultimapScoreRange 1 --outFilterMatchNminOverLread 0 --outFilterMismatchNmax 3 --outFilterMismatchNoverLmax 0.1 --quantMode TranscriptomeSAM). To calculate the reads on the CDS, HTseq (version 0.6.1p1; --stranded=no --mode=intersection-nonempty -r pos) was used with the customized RefSeq reference (longest isoform among transcripts of hg19). For additional analysis, uniquely mapped reads were chosen and further analyzed with the R package deltaTE. A custom python script was used to create the plots on the basis of the deltaTE results.

### RNA-seq library construction and analysis

Total cellular RNA was extracted as described above, and the RNA-seq library was constructed using a TruSeq Stranded Total RNA Kit (Illumina). Sequencing was performed with the NovaSeq 6000 platform (Illumina). The raw reads were trimmed using TrimGalore (v0.6.7), and index building and alignment were then performed using HISAT2 (v2.2.1). FeatureCounts (v2.0.3) was applied to generate read counts, which were then analyzed using R (v4.2.0) in RStudio. Comparisons between two samples were performed using DESeq2 (v1.38.2). The resulting list of significantly differentially expressed genes was used to identify corresponding changes in gene sets by performing DAVID (v2021) gene ontology analysis. The enrichment of a specific gene set between two samples was assessed by performing pre-ranked GSEA (v4.2.3).

### m^7^ GTP pulldown assay

Harvested cells were lysed in NETN lysis buffer (20 mM Tris-HCl [pH 8.0], 100 mM NaCl, 0.5% Nonidet P-40, and 1 mM EDTA), and the lysates were centrifuged at 15,000 × *g* for 20 min at 4 °C. The resulting supernatants (1 mg of protein in 1 ml) were incubated with 20 μl of protein A/G-agarose beads (Thermo Fisher) for 1 h at 4 °C with rotation to remove nonspecific bead-binding proteins. The supernatants were then incubated with 25 μl of m^7^ GTP Sepharose beads (Jena Bioscience) for 1 h at 4 °C with rotation, after which the beads were washed five times with lysis buffer and boiled with 2x Laemmli sample buffer. After brief centrifugation to remove debris, the samples were subjected to immunoblot analysis.

### Soft agar colony formation assay

The cell growth medium (2×) was mixed with an equal volume of 1% noble agar (Sigma), and portions (1.5 ml) of the resulting mixture were allowed to solidify in the wells of six-well plates. A similar preparation of 0.35% agar mixed with an equal volume of growth medium containing 5 × 10^3^ cells was added on top of the 0.5% agar layer. The growth medium (1 ml) was then added on top of the two agar layers and replenished twice a week throughout the 3-week culture period. The resulting colonies were fixed and stained for 2 h with 4% paraformaldehyde and 0.005% crystal violet (Sigma) in PBS, and the wells were washed multiple times with PBS before imaging for quantification and analysis.

### Transwell migration assay

Tet-ON DDIT4 92.1 and OCM1 cells were cultured in the presence or absence of Dox (1 μg/ml) for 72 h prior to trypsinization and resuspension in serum-free medium (SFM) containing 0.1% bovine serum albumin (BSA). The resulting cell suspensions (200 μl containing 2 × 10^5^ cells) were added to transwell inserts (SPL) in SFM containing Dox (1 μg/ml), and complete medium containing serum and Dox (1 μg/ml) was added underneath the inserts. After 24 h, the transwell inserts were fixed with 4% paraformaldehyde (Sigma) in PBS for 10 min. The cells remaining on the upper surface of the insert were removed using a cotton swab, and the lower surface of the insert was subsequently mounted onto a glass slide (Marienfeld) with a mounting solution containing DAPI (Vectorshield). The edges were sealed with clear nail polish, and at least 3 random regions per insert were subsequently imaged with an inverted fluorescence microscope (Zeiss).

### Cell proliferation assay

Cell suspensions (100 μl containing 1 × 10^3^ cells) were added to the wells of 96-well plates and cultured for various durations. EZ-CytoX reagent (DoGenBio) was added to the medium 2 h prior to measuring the absorbance at 450 nm with a plate reader (Molecular Devices). Measurements were performed daily, and signals were normalized to those of wells containing growth medium without cells.

### Luciferase reporter assay

The region of the human DDIT4 promoter, including the ORF (−1.5 kb ~ +1 kb relative to the TSS), was amplified from genomic DNA isolated from OCM1 cells and cloned and inserted into the pGL3 basic vector via the KpnI restriction enzyme. Then, each well of 293AD cells cultured in 12-well plates was cotransfected with 1 μg of vector control or FLAG-YAP 5SA or FLAG-YAP 5SA-S94A, 200 ng of pGL3 firefly luciferase plasmid containing either the DDIT4 promoter or an artificial 8X-tandem TEAD-binding sequence (8XTBS), and 20 ng of pRL Renilla plasmid using PEI. After 48 h, the cells were harvested, and reporter activity was measured using the Dual-Luciferase Reporter Assay Kit (Promega) and a microplate reader (Berthold Technologies).

### Xenograft tumor formation

Cell suspensions (3 × 10^6^ Tet-ON DDIT4 92.1 cells or 1 × 10^6^ Tet-ON DDIT4 OCM1 cells in 100 μl of growth medium) were mixed with an equal volume of Matrigel (BD Biosciences), and the mixture was injected subcutaneously into the left flank of 6-week-old female athymic nude mice (CanN.Cg-Foxn1 nu/CrlOri strain, OrientBio). Tumor growth was measured twice a week with a caliper, and tumor volume was calculated as (length × width^2^)/2. The mice were treated orally with doxycycline (0.5 mg/ml), which was prepared as a 10 mg/ml stock solution in deionized water, sterilized by filtration, diluted 1:20 in drinking water, and replenished weekly.

### Quantification and statistical analysis

Graphs were drawn with GraphPad Prism (ver. 9.3.1) software. Statistical analysis was performed using a two-tailed, unpaired Student’s *t* test with a 95% confidence interval or standard one-way ANOVA. A *p* value of <0.05 was considered statistically significant. Unless indicated otherwise, all statistically relevant experiments were performed with at least two technical replicates and at least three biological replicates.

## Results

### YAP overcomes the serum starvation-induced suppression of mRNA translation

As noted earlier, some studies have reported that Hippo pathway inactivation or YAP/TAZ activation promotes mTORC1 activation^35^. However, given that the mTORC1 effectors S6K1 and 4E-BP modulate protein translation in eukaryotic cells, we tested whether YAP is sufficient to drive mRNA translation, an aspect of this crosstalk that has not previously been explored. To measure relative translation levels, we employed the SUnSET method^36^, in which general translation can be assessed in cells pulsed with puromycin (which mimics tyrosyl-tRNA) using a puromycin-selective antibody.

**Fig. 1:**
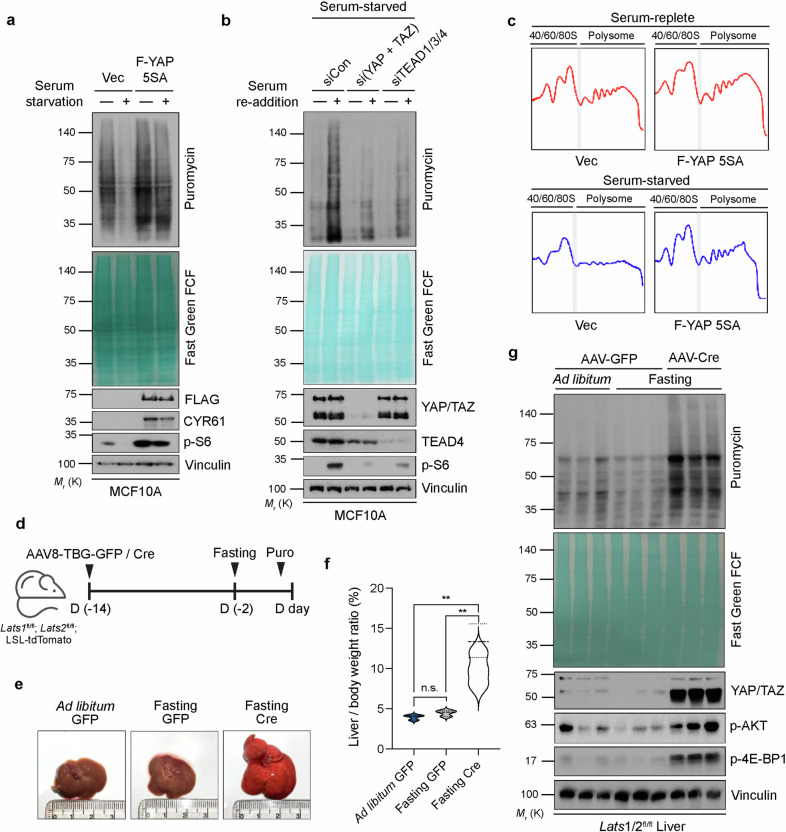
YAP overcomes serum starvation-induced suppression of mRNA translation. **a** MCF10A cells stably expressing the vector control or FLAG epitope (F)-tagged YAP 5SA construct were serum starved (or not) overnight and exposed briefly to puromycin for assessment of relative translation levels. The cell lysates were subjected to immunoblot analysis with antibodies against the indicated proteins. CYR61, a canonical YAP/TAZ–TEAD target gene, was used as a positive control for the overexpression of YAP 5SA. The blot membrane was also stained with Fast Green FCF. **b** MCF10A cells transiently transfected with a control (Con) siRNA or with siRNAs that target both YAP and TAZ or TEAD1/3/4 were serum starved overnight, incubated for 1 h with or without serum, and analyzed as described in (**a**). **c** Polysome profiling plots for 293AD cells stably expressing the control vector or YAP 5SA that had been serum starved (or not) overnight. Peaks corresponding to ribosome subunits (40S and 60S), monosomes (80S), and polysomes are indicated. **d** Experimental scheme for the measurement of relative translation in vivo. *Lats1/2*^fl/fl^;LSL-tdTomato mice (*n* = 3 mice per group) were injected intraperitoneally with AAV8-TBG-GFP or AAV8-TBG-Cre, starved (or not) of chow for 48 h, and injected intraperitoneally with puromycin 30 min before analysis. **e** Representative images of the excised livers from the mice in (**d**). The redness of the livers from Cre-injected mice reflects efficient recombination-dependent expression of tdTomato. **f** Violin plots of the mouse liver/body weight ratio in (**d**). The centerline indicates the median, the dotted lines above and below the median represent the upper and lower quartiles, and the boundaries denote the maximum and minimum values. **p < 0.005; n.s. not significant (one-way ANOVA). **g** Lysates of the livers from the mice in (**d**) were analyzed as described in (**a**).

Forced expression of YAP 5SA (a constitutively active form due to mutations in LATS phosphorylation sites) alone in MCF10A human mammary epithelial cells did not substantially affect basal translation levels but readily induced the expression of CYR61 (encoded by the *CCN1* gene), a canonical YAP/TAZ target (Fig. 1a). We therefore tested several experimental conditions that have been shown to affect the Hippo–YAP/TAZ pathway or global translation. Among the conditions tested, both serum starvation and osmotic stress attenuated translation (Supplementary Fig. 1a, b), whereas acute disruption of actomyosin tension by latrunculin B treatment had no marked effect (Supplementary Fig. 1d). Notably, all three stresses inhibited YAP, as indicated by the reduction in the unphosphorylated, fast-migrating band on the phos-tag gel. Intriguingly, YAP 5SA attenuated the restriction of translation induced by serum starvation (Fig. 1a), which was consistent across diverse cell lines, including RPE-1 (human retinal pigment epithelial cells), HaCaT (human keratinocytes), AML12 (murine hepatocytes), and 293AD (human embryonic kidney cells) (Supplementary Fig. 2). However, osmotic stress-induced suppression of translation^37^ could not be restored by YAP 5SA (Supplementary Fig. 1c), suggesting that YAP selectively regulates serum-mediated translation. Notably, osmotic stress leads to the exclusion of TEADs from the nucleus, potentially explaining why YAP 5SA alone fails to rescue translation^38^. Conversely, knockdown of both YAP and TAZ or TEADs greatly attenuated the restoration of translation and S6 phosphorylation induced by serum stimulation in serum-starved cells (Fig. 1b). In addition, treatment of serum-starved cells with conditioned medium obtained from YAP 5SA-expressing cells did not affect translation, suggesting that YAP-induced translation has a cell-autonomous effect (Supplementary Fig. 1e). An increase in the cellular content of polysomes characterizes active translation. We therefore performed polysome profiling to determine whether YAP 5SA might increase the polysome content in serum-starved cells. Whereas polysome peaks were completely abolished in response to serum starvation in control cells, polysomes were effectively maintained in serum-starved cells expressing YAP 5SA (Fig. 1c).

We next tested whether the induction of YAP/TAZ-mediated translation is conserved in vivo. To activate YAP/TAZ in vivo, we deleted *Lats1* and *Lats2* in the liver by injecting mice harboring floxed alleles of these genes (*Lats1/2*^fl/fl^;LSL-tdTomato) with a liver-selective adeno-associated virus (AAV8-TBG) encoding either GFP (control) or Cre recombinase (Fig. 1d). The mice were subsequently subjected to fasting as a means for nutrient deprivation in vivo, as fasting has been demonstrated to suppress mTORC1 activity in the liver^39^. Although fasting alone did not induce a marked change in the gross morphology or weight of the liver (Fig. 1e, f), lysates obtained from these puromycin-injected mice presented a general decrease in translation (Fig. 1g). In contrast, compared with the livers of fasted control mice, the livers of fasted *Lats1/2*-knockout mice were markedly increased in both size and weight. Notably, liver lysates from fasted knockout mice presented pronounced increases in translation as well as in the phosphorylation of the mTORC1 effector 4E-BP (Thr^37/41^) and Akt (Ser^473^), which promote mTORC1 activity in response to growth factor signals (Fig. 1g). These results are consistent with our earlier findings that YAP 5SA restores mTORC1 inhibition and translation restriction in serum-starved cells in vitro.

### YAP induces the translation of translation-related genes upon serum starvation

**Fig. 2:**
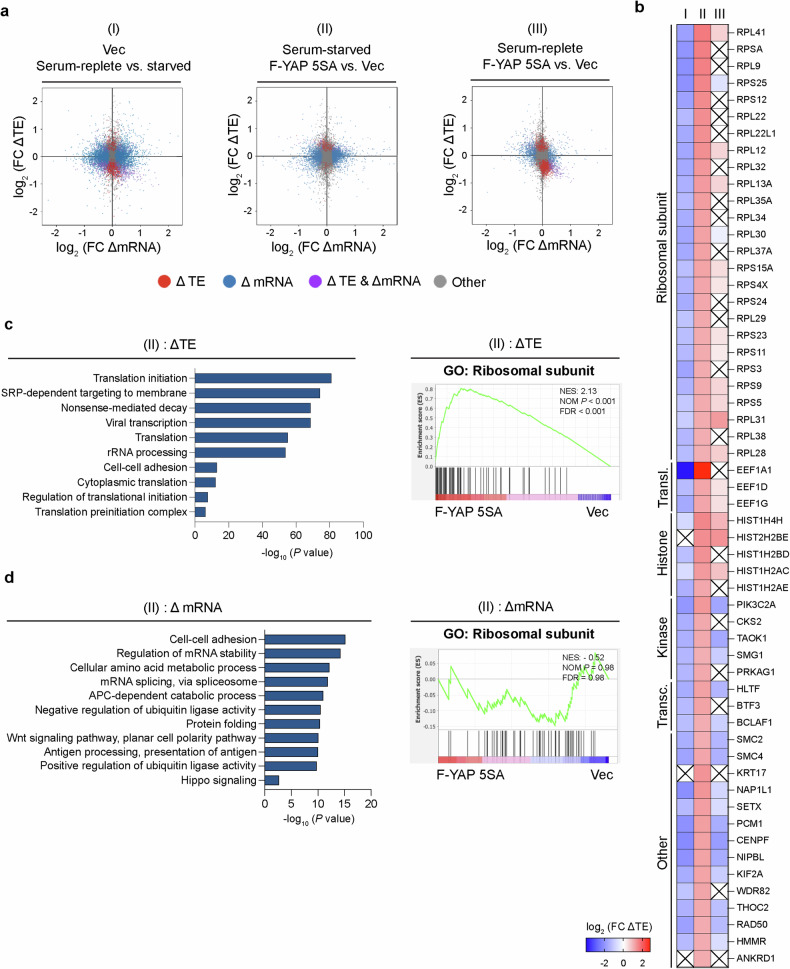
YAP induces translation of translation-related genes upon serum starvation. **a** Scatter plots of the relative log_2_-transformed fold change in translation efficiency (ΔTE) or mRNA abundance (ΔmRNA), or both parameters, as determined using Ribo-seq and RNA-seq analysis for 293AD cells stably expressing vector control or YAP 5SA, with the cells either being serum starved overnight or maintained in the presence of serum (comparisons are denoted I, II, III for future reference, as indicated). Each dot represents a single gene, and those genes with a corresponding FDR of <0.05 are indicated in orange (ΔTE), blue (ΔmRNA), or purple (ΔTE & ΔmRNA). **b** Heatmap illustration of genes with significant (FDR of <0.05) ΔTE in (II) with a log_2_(fold change) >1, grouped by their known functional roles. The crosses with boxes (“X”) denote nonsignificant (FDR >0.05) comparisons. **c** (Left) GO analysis performed using DAVID for Ribo-seq data from serum-starved 293AD cells stably expressing the vector control or YAP 5SA. The top 10 enriched terms for the significantly upregulated genes are listed with the corresponding −log_10_(*p* values). (Right) GSEA for the GO term “ribosomal subunit”. NES normalized enrichment score, NOM *P* nominal *p* value, FDR false discovery rate. **d** (Left) GO analysis of RNA-seq data from serum-starved 293AD cells stably expressing the vector control or YAP 5SA was performed using DAVID. The top 10 enriched terms for the significantly upregulated genes as well as the “Hippo signaling” term are listed with the corresponding −log_10_(*p* values). (Right) GSEA for the GO term “ribosomal subunit”. NES normalized enrichment score, NOM *P* nominal *p* value, FDR false discovery rate.

To gain further insight into the translatome governed by active YAP, we performed Ribo-seq on both YAP 5SA-expressing and control cells subjected to serum starvation (Fig. 2a, b and Supplementary Tables 1 and 2). Notably, translation efficiency (ΔTE) generally decreased due to serum starvation (I), whereas ΔTE generally increased in serum-starved cells expressing YAP 5SA(II) (Fig. 2a). Conversely, the overall ΔTE between YAP 5SA-expressing and control cells was not particularly different under serum-replete conditions (III) (Fig. 2a). Intriguingly, examination of the genes with a significant increase in ΔTE for (II) revealed several ribosomal or translation-related genes (Fig. 2b). To gain insight into which cellular processes were the most altered, Gene Ontology (GO) analysis was performed. As expected, translation-related processes were more enriched in the YAP 5SA-expressing cells than in the control cells under serum starvation conditions (Fig. 2c). Indeed, gene set enrichment analysis (GSEA) of a selective “ribosomal subunit” gene set with genes significantly affected by Ribo-seq also revealed significant enrichment in the serum-starved YAP 5SA-expressing cells (Fig. 2c). In contrast, GO and GSEA of the RNA-seq data from the same samples did not reveal a marked increase in the transcription of translation-related genes in the serum-starved YAP5SA-expressing cells relative to the control cells (Fig. 2d). Notably, neither serum starvation nor YAP 5SA overexpression noticeably altered the ribosome-protected fragment (RPF) length distribution or codon-relevant metrics (Supplementary Fig. 3a, b), implying that the translational changes observed above are not attributed to discrepancies in ribosome binding or codon usage. These results collectively indicate that YAP 5SA actively promotes the translation (but not necessarily transcription) of translation- or ribosome-related genes selectively in serum-starved cells.

### YAP overrides serum starvation-induced mTORC1 suppression to drive cap-dependent translation

**Fig. 3:**
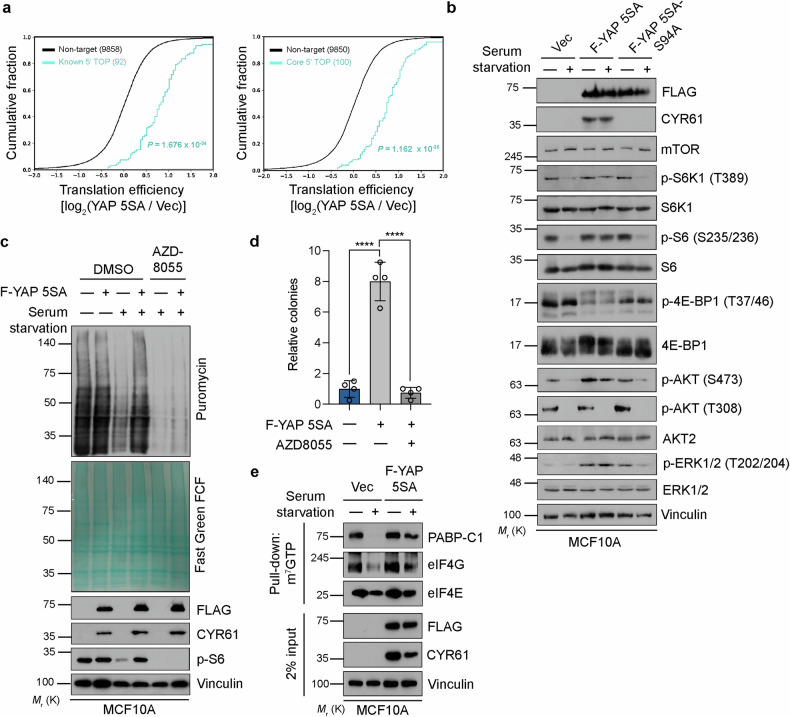
YAP overrides serum starvation-induced mTORC1 suppression to drive cap-dependent translation. **a** Cumulative distribution of translation efficiency [log_2_(F-YAP 5SA/Vec)] determined for the Ribo-seq samples from Fig. 2. The number of known and “core” 5′TOP-containing transcripts^40^ significantly shifted to the right. **b** MCF10A cells stably expressing the vector control, YAP 5SA, or YAP 5SA-S94A were serum starved (or not) overnight and then subjected to immunoblot analysis of the indicated mTORC1 signaling-related proteins. **c** MCF10A cells stably expressing the vector control or YAP 5SA were serum starved (or not) of serum and exposed to the mTOR inhibitor AZD8055 (1 µM) for the final 2 h before analysis, as shown in Fig. 1a. **d** Relative anchorage-independent colony formation quantification of MCF10A cells stably expressing the control vector or YAP 5SA maintained in the presence of AZD8055 (1 µM) for 3 weeks. The data are presented as the means ± s.e.m. (*n* = 4 independent replicates). ****p < 0.0001 (unpaired Student’s *t* test). **e** MCF10A cells stably expressing the vector control or YAP 5SA were serum starved (or not) overnight, after which the cell lysates were subjected to a pull-down assay with m^7^GTP-conjugated beads. The precipitates as well as a portion (2%) of the original lysates were then subjected to immunoblot analysis of the indicated proteins.

mTORC1 primarily regulates the translation of transcripts harboring a 5′TOP, which includes many encoding components of the translational machinery, including genes upregulated by YAP 5SA under serum starvation conditions (*RPL41*, *EEF1A1*, etc.) (Fig. 2b). We therefore examined the translation of 5′TOP-containing transcripts in our Ribo-seq dataset. A cumulative distribution plot of the Ribo-seq results revealed a significant increase in translation efficiency for known and core^40^ 5′TOP-containing transcripts in YAP 5SA-expressing cells compared with control cells under the context of serum starvation (Fig. 3a).

Given that YAP 5SA restored the general translational output and S6 phosphorylation in serum-starved cells and that mTORC1 plays a dominant role in the regulation of general translation dynamics, we next comprehensively analyzed mTORC1 signaling. Consistent with the observed pattern of S6 phosphorylation, the patterns of S6K1 and 4E-BP were also rescued by YAP 5SA but not by the TEAD binding-defective mutant YAP 5SA-S94A (Fig. 3b). Moreover, YAP 5SA increased ERK1/2 (extracellular signal-regulated kinase) phosphorylation at Thr^202^/Tyr^204^ and AKT phosphorylation at Ser^473^ (target of mTOR) but not at Ser^308^ (target of PDK1) in serum-starved cells. To confirm that mTORC1 is responsible for YAP-induced translation, we examined the effects of the mTORC1 inhibitor AZD8055. Indeed, AZD8055 abolished YAP 5SA-induced S6 phosphorylation, the associated induction of general translation (Fig. 3c) and cellular transformation as assessed by colony formation in soft agar (Fig. 3d).

Finally, serum starvation abrogated the association of the 7-methyl-GTP (m^7^GTP) 5′ cap-binding protein eIF4E with the 3′ poly(A)-binding protein PABP-C1, but this association, which mediates mRNA circularization, a key process for efficient translation initiation^41^, was restored by YAP 5SA expression (Fig. 3e). These results collectively indicate that YAP 5SA drives the translation of 5′TOP-containing transcripts selectively in serum-starved cells, thereby increasing the cellular pool of components of the translational machinery to upregulate general translation.

### Upregulation of DDIT4 transcription upon serum starvation is repressed by YAP

**Fig. 4:**
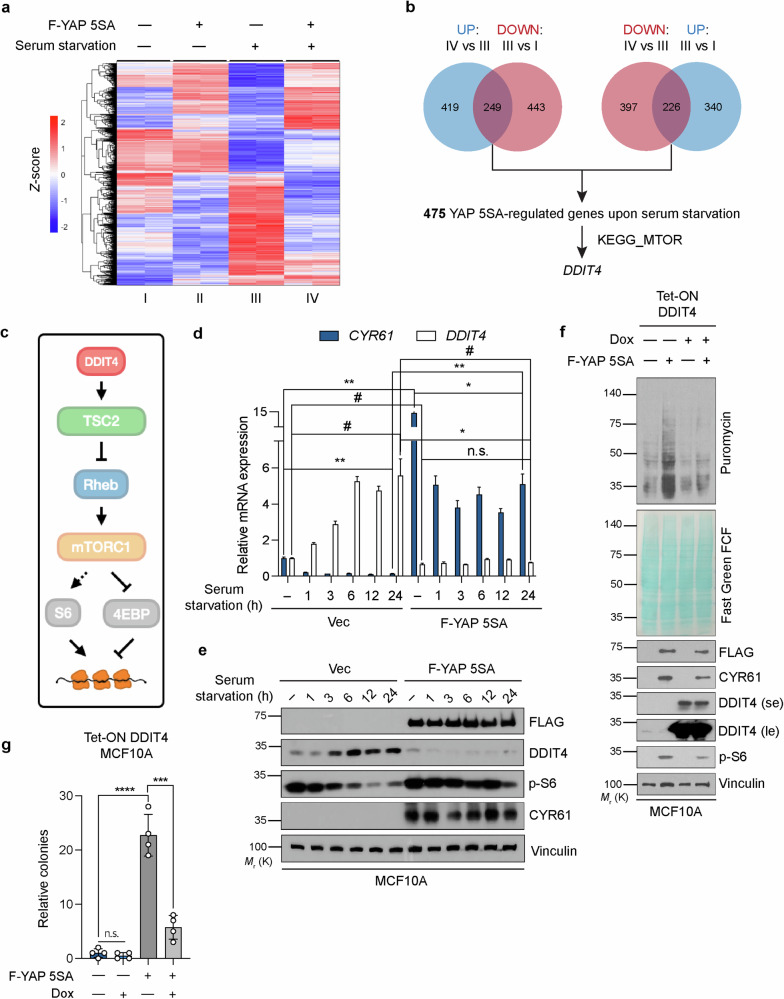
Upregulation of DDIT4 transcription upon serum starvation is repressed by YAP. **a** Heatmap of *Z* scores for RNA-seq expression profiles of MCF10A cells stably expressing the control vector or YAP 5SA subjected (or not) to overnight serum starvation. The samples are labeled “I to IV” for easy reference. **b** Venn diagrams showing the number of overlapping genes for the samples in (**a**) after filtering according to a *p* value of <0.05 and an absolute fold change of >3. The left Venn diagram shows the overlap of genes upregulated by YAP 5SA under serum starvation conditions with those downregulated by serum starvation in control cells. The right Venn diagram shows genes that behave in the opposite manner. The overlapping genes from each Venn diagram were combined to identify a gene signature regulated by YAP 5SA in response to serum starvation. These genes were then filtered relative to the KEGG_MTOR signature to identify any genes that encode proteins related to mTOR signaling. Only *DDIT4* was identified using this analysis. **c** Schematic model illustrating the signaling axis by which DDIT4 ultimately regulates mRNA translation. **d** Quantitative reverse transcription and polymerase chain reaction (qRT‒PCR) analysis of the mRNA abundance of *DDIT4* and *CYR61* in MCF10A cells stably expressing the control vector or YAP 5SA subjected to serum starvation for the indicated times. The bar graphs represent the means of technical replicates (*n* = 3 independent replicates). Data are presented as the means ± s.e.m. (*n* = 3). The symbols * and ^#^ indicate comparisons with *CYR61* and *DDIT4*, respectively. */^#^*p* < 0.05, **p < 0.005; n.s. not significant (unpaired Student’s *t* test). **e** Immunoblot analysis of the samples in (**d**). **f** MCF10A cells harboring a doxycycline (Dox)-inducible DDIT4 expression construct (Tet-ON DDIT4) and stably expressing the vector control or YAP 5SA were serum starved and treated with doxycycline (1 µg/ml) before analysis, as shown in Fig. 1a. **g** Relative anchorage-independent colony formation quantification of Tet-ON DDIT4 MCF10A cells stably expressing the control vector or YAP 5SA and maintained in the absence or presence of doxycycline for 3 weeks. The data are presented as the means ± s.e.m. (*n* = 4 independent replicates). ***p < 0.0005, ****p < 0.0001; n.s. not significant (unpaired Student’s *t* test).

Given that YAP 5SA selectively increases global translation in serum-starved cells, we hypothesized that this effect is likely achieved by YAP regulating the expression of particular genes that exhibit opposite patterns of regulation under conditions of serum starvation. We therefore compared the expression profiles of YAP 5SA-expressing and control MCF10A cells under normal or serum-starved conditions (Fig. 4a and Supplementary Table 3). Among the genes significantly upregulated by YAP 5SA in serum-starved cells, 249 genes overlapped with those downregulated by serum starvation in control cells. In addition, 226 genes behaved in the opposite manner (Fig. 4b). Filtering of the total 475 overlapping genes relative to an established list of genes related to mTOR signaling (KEGG_MTOR) yielded only one gene, DNA damage-inducible transcript 4 (*DDIT4*) (Fig. 4b and Supplementary Fig. 4a). Notably, *DDIT4* was identified among the genes downregulated by YAP 5SA, suggesting that YAP mediates its transcriptional repression. In addition, none of the mTOR-upstream GATOR1/2 complex genes were significantly affected by either YAP 5SA or serum starvation (Supplementary Fig. 4b, c).

We previously identified *DDIT4* as a gene that is transcriptionally repressed by YAP/TAZ in a manner dependent on recruitment of the NuRD complex^6^. Notably, *DDIT4* induction in response to hypoxia and subsequent inactivation of mTOR signaling (Fig. 4c) were completely suppressed by YAP 5SA^6^, establishing *DDIT4* as a bona fide link between YAP/TAZ and mTOR signaling. In addition to hypoxia and other cellular stressors^29^, nutrient deprivation also induces *DDIT4* expression^28^. Given that serum starvation suppressed both translation and mTORC1 activity, we tested whether such prolonged treatment might induce *DDIT4* expression and whether YAP 5SA abrogates this effect. Consistent with previous findings^28^, *DDIT4* expression at both the mRNA (Fig. 4d and Supplementary Fig. 5a) and protein (Fig. 4e) levels increased gradually concomitant with a decrease in S6 phosphorylation in cells subjected to serum starvation. Moreover, this effect of serum starvation on *DDIT4* expression was markedly suppressed by YAP 5SA (Fig. 4d and Supplementary Fig. 5a) but was largely unaffected by YAP 5SA-S94A (Supplementary Fig. 5b). This finding indicates that this effect was mediated by the binding of YAP to TEADs. Consistent with these results, *DDIT4* expression was increased in cells depleted of YAP, TAZ or TEADs (Supplementary Fig. 5c) and those treated with the YAP-TEAD inhibitor verteporfin (Supplementary Fig. 5d). Finally, only YAP 5SA, but not YAP 5SA-S94A, readily induced the luciferase activity of the DDIT4 promoter sequence including the ORF that was cloned and inserted into a reporter plasmid (Supplementary Fig. 5e).

To test whether DDIT4 is indeed responsible for the suppression of mTORC1 activity in serum-starved cells and whether the loss of DDIT4 is responsible for the activation of mTORC1 in response to YAP 5SA expression, we next examined whether forced DDIT4 expression is sufficient to block translation. Consistent with the previously demonstrated mTOR-suppressive role of DDIT4^29,42^, the induction of DDIT4 expression greatly attenuated the YAP 5SA-mediated increase in translation and S6 phosphorylation in serum-starved cells (Fig. 4f). We also evaluated whether DDIT4 might inhibit cell transformation via YAP 5SA. Indeed, soft agar colony formation assays revealed that forced DDIT4 expression significantly inhibited YAP 5SA-induced colony formation (Fig. 4g).

### DDIT4 suppresses the tumorigenicity of GPCR-mutant uveal melanoma cells

Given that YAP-TEAD mediates the transcriptional repression of *DDIT4* in the presence of serum, we sought to determine a physiologically relevant context in which cells are unresponsive to such growth factors. Uveal melanomas (UMs) frequently harbor mutations in *GNAQ* or *GNA11*^33,34^, both of which encode G protein subunits. These mutations result in constitutive activation of the corresponding G proteins, thereby rendering cells unresponsive to the external serum status^43^. The Hippo pathway is regulated by G protein-coupled receptor signaling^43^, and YAP/TAZ are required for the tumorigenic properties of G protein-mutated UMs^44^. Therefore, we hypothesized that the regulation of translation by the YAP/TAZ–DDIT4–mTORC1 axis might play a key role in this context.

**Fig. 5:**
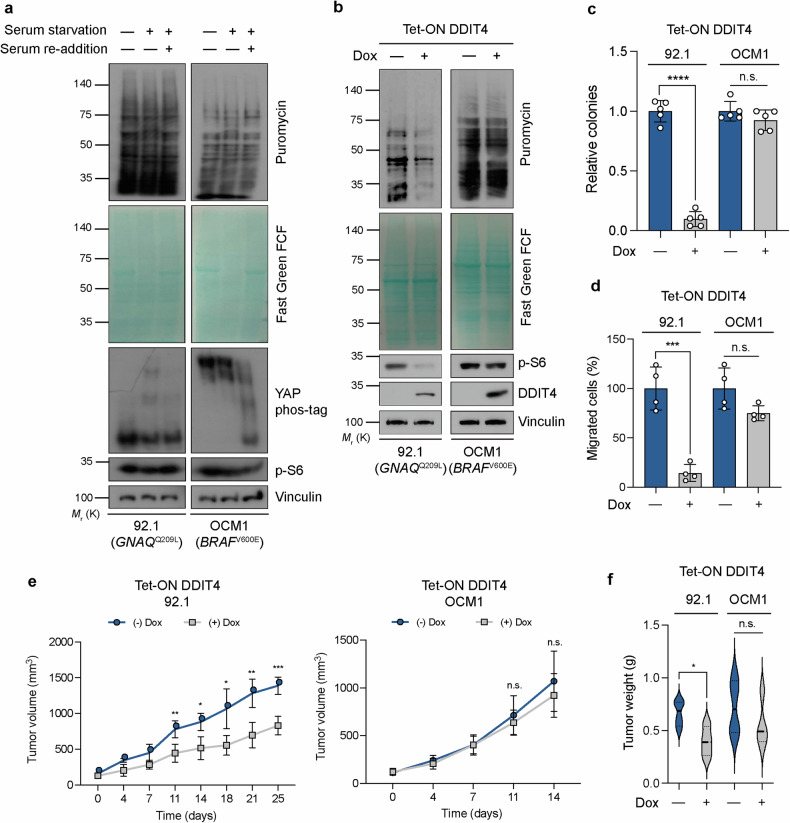
DDIT4 suppresses the tumorigenicity of YAP-dependent uveal melanoma cells. **a** *GNAQ*^Q209L^-mutant 92.1 and *BRAF*^V600E^-mutant OCM1 uveal melanoma cells were serum starved (or not) overnight and then restimulated (or not) with serum for 1 h. The samples were then subjected to analysis, as shown in Fig. 1a, as well as to YAP Phos-tag gel analysis. **b** Tet-ON DDIT4 92.1 and OCM1 cells were incubated in the absence or presence of doxycycline (Dox, 1 µg/ml) for 24 h and then analyzed as described in (**a**). **c** Relative anchorage-independent colony formation of Tet-ON DDIT4 92.1 or OCM1 cells maintained in the absence or presence of doxycycline for 3 weeks. The data are presented as the means ± s.e.m. (*n* = 5 independent replicates). ****p < 0.0001; n.s. not significant (unpaired Student’s *t* test). **d** Relative transwell migration quantification of Tet-ON DDIT4 92.1 and OCM1 cells in the absence or presence of doxycycline. The data are presented as the means ± s.e.m. (*n* = 4 independent replicates). ***p < 0.0005; n.s. not significant (unpaired Student’s *t* test). **e** Time course of xenograft tumor volume in nude mice injected with Tet-ON DDIT4 92.1 or OCM1 cells and treated (or not) with doxycycline (0.5 mg/ml) in the drinking water. The data are presented as the means ± s.e.m. (*n* = 4 mice per group). *p < 0.05, **p < 0.005, ***p < 0.0005; n.s. not significant (unpaired Student’s *t* test). **f** Weights of excised xenograft tumors from the mice in (**e**) at the end points (25 days or 14 days after Tet-ON DDIT4 92.1 or OCM1 cell injection, respectively). The data are presented as the means ± s.e.m. (*n* = 4 mice per group). *p < 0.05; n.s. not significant (unpaired Student’s *t* test).

Consistent with previous findings^44^, YAP was constitutively dephosphorylated (active) in *GNAQ*-mutant 92.1 UM cells (Fig. 5a). In contrast, it was predominantly phosphorylated in *BRAF*-mutant OCM1 UM cells. Although translation and S6 phosphorylation were largely unaffected by serum starvation or restimulation in both cell lines, YAP phosphorylation was greatly reduced by serum restimulation in OCM1 cells. In other words, despite YAP activation in OCM1 cells, translation was not increased, providing further support for the notion that *BRAF*-mutant UMs are not particularly dependent on YAP/TAZ^44^. Given that YAP is constitutively activated in 92.1 cells, *DDIT4* is expected to be constitutively suppressed, and mTORC1 activity is expected to be unrestrained. To test this hypothesis, we generated stable 92.1 and OCM1 cell lines harboring a doxycycline-inducible DDIT4 expression system. Both translation and S6 phosphorylation were markedly inhibited by doxycycline treatment in the Tet-ON DDIT4 92.1 cells, but the effects were negligible in the Tet-ON DDIT4 OCM1 cells (Fig. 5b). Similarly, treatment with the mTOR inhibitor AZD8055 attenuated translation and S6 phosphorylation to a markedly greater extent in the former cells than in the latter (Supplementary Fig. 6a).

Given that we established the importance of DDIT4 and mTOR in the regulation of translation in G protein-mutant UMs with constitutive YAP activation, we performed several assays to assess the tumorigenic potential of these cells. We first measured relative cell proliferation. As expected, 92.1 cells exhibited far greater sensitivity to AZD8055-induced growth retardation than OCM1 cells did (Supplementary Fig. 6b). We next measured anchorage-independent growth in soft agar and found that forced DDIT4 expression or AZD8055 treatment significantly attenuated colony formation (Fig. 5c and Supplementary Fig. 6c) and transwell migration (Fig. 5d) in 92.1 cells. Finally, we evaluated tumorigenic capacity in vivo via the subcutaneous injection of Tet-ON DDIT4 92.1 or OCM1 cells into the flanks of nude mice. Consistent with our in vitro findings, treatment of the mice with doxycycline significantly reduced tumor volume and weight exclusively in the mice injected with Tet-ON DDIT4 92.1 cells (Fig. 5e, f). Collectively, these data indicate that G protein-mutant UMs with constitutive YAP activation are more dependent on DDIT4–mTOR-mediated regulation of translation for their tumorigenic properties than are *BRAF*-mutant UMs with inactive YAP.

## Discussion

Studies have shown a correlation between YAP/TAZ and mTORC1 in various tissues of multiple organisms^12,35,45^. However, the relationship of YAP and TAZ has not been previously explored with respect to translation, an anabolic process essential for cell growth that ultimately results in mTORC1 activation. We next assessed the net translational output in cells expressing YAP 5SA and discovered that it largely reversed the suppression of translation induced by serum starvation. Moreover, our Ribo-seq data revealed that active YAP selectively induced the translation of ribosome- and translation-related genes in serum-starved cells. Interestingly, this process is dependent on mTORC1, which accounts for the translation of most mRNAs. In particular, mTORC1 regulates the translation of 5′TOP-containing mRNAs, which encode components of the translational machinery, such as ribosomal proteins and translation factors. Our results thus indicate that active YAP drives the de novo synthesis of ribosomes and translation factors, leading to increased global translation, thereby reversing the translational deficit caused by serum starvation.

**Fig. 6:**
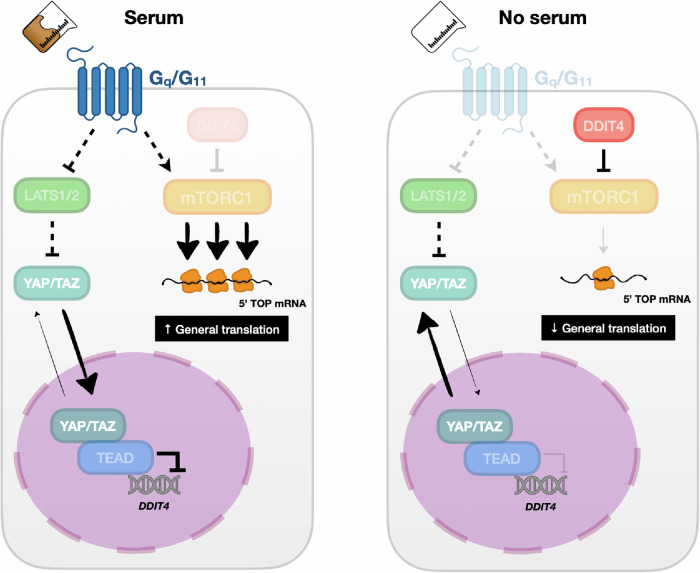
Proposed model for the regulation of translation by serum in a manner dependent on the YAP/TAZ–TEAD–DDIT4–mTORC1 axis. (Left) In cells with an adequate supply of nutrients and growth factors (serum), G proteins such as G_q_/G_11_ become activated, resulting in LATS1/2 inactivation and the consequent dephosphorylation of YAP/TAZ, which then translocate to the nucleus and bind to cognate TEAD transcription factors to activate the transcription of downstream target genes. However, some genes, such as *DDIT4*, are transcriptionally repressed by YAP/TAZ. Given that DDIT4 suppresses mTORC1 activity via TSC1/2, downregulation of DDIT4 by YAP/TAZ promotes mTORC1 activation, which ultimately leads to increased cap-dependent translation, especially of 5′TOP-containing mRNAs that encode components of the translational machinery. (Right) Conversely, YAP/TAZ are inactive under nutrient-poor conditions, resulting in high DDIT4 expression, suppression of mTORC1 activity, and inhibition of translation. Forced YAP activation in serum-starved cells is sufficient to restore translation to levels characteristic of serum-replete conditions.

We found that the expression of DDIT4, an important negative regulator of mTORC1, was induced by serum starvation and that YAP attenuated this effect. Forced DDIT4 expression was sufficient to suppress YAP-induced translation in serum-starved cells and YAP-induced anchorage-independent colony formation. In pursuit of a suitable physiological context independent of serum but dependent on YAP, we assessed whether the YAP–DDIT4 translation axis might operate in G protein-mutated uveal melanoma, which was previously shown to be dependent on YAP-TEAD. As expected, neither translation nor S6 phosphorylation was affected by serum starvation in these cells. Importantly, however, forced DDIT4 expression strongly suppressed both these processes and the tumorigenic properties of these *GNAQ*-mutant UM cells. In contrast, forced DDIT4 expression did not substantially affect translation, S6 phosphorylation, or colony formation in *BRAF*-mutant UM cells, highlighting the importance of DDIT4, which is dependent on YAP, specifically in G protein-mutant cells. These data therefore provide a promising rationale for targeting DDIT4 or mTORC1 in the treatment of YAP-driven malignancies. Collectively, our results define a direct linear model in which YAP/TAZ–TEAD activate mTORC1 to promote global translation (Fig. 6).

Although neither translation nor ribosome-related processes were the most enriched by YAP at the mRNA level in our study, these processes are often among the most significantly upregulated processes in YAP-active contexts, including genetic ablation of upstream negative regulators in vivo^46,47^. We postulate that YAP indirectly influences translation in a feed-forward manner given that YAP transcribes many genes essential for cell proliferation and survival^4,8^. For example, several studies have demonstrated that YAP induces (transcriptionally or posttranscriptionally) the expression of MYC^46,48^, a transcription factor that is widely regarded as a master regulator of ribosome biogenesis^49^. MYC not only binds to Pol II to promote the expression of ribosomal proteins (RPs) but also binds to Pol I and III to promote the transcription of rRNAs, which together with RPs constitute the ribosome^50^. Therefore, future studies dedicated to exploring the relationship between YAP and MYC at the translational level will not only provide great mechanistic insight but also hold enormous clinical implications, as *MYC* is frequently activated in diverse cancers^51^.

The direct upstream negative regulators of YAP/TAZ, LATS1/2, are rarely mutated or lost in human cancers^52^, making it difficult to generalize the concepts introduced in our study using primarily hyperactive YAP mutants. Nevertheless, mutations in other upstream components are more frequently observed, rendering YAP highly active. For example, oncogenic mutations in *KRAS* drive YAP-dependent pancreatic ductal adenocarcinoma^53^ and lung cancer^54^. Notably, the role of oncogenic Ras in driving protein translation has been well documented^55^. Additionally, loss-of-function mutations in *NF2* are often found in 19–50% of malignant mesotheliomas^56^ and 18–22% of renal cell carcinomas^57^. NF2 is believed to be critical for LATS activation to the extent that NF2 deletion can phenocopy LATS deletion in cultured cells and mice^58^. Moreover, the loss of NF2 renders cells unresponsive to serum in terms of YAP phosphorylation^59^, suggesting that NF2 is involved in the YAP/mTOR-mediated translation mechanism presented here. Finally, we demonstrated that G protein-mutant uveal melanomas, which are driven by YAP/TAZ, are highly dependent on mTOR-mediated translation. In contrast, *BRAF*-mutant uveal melanomas likely harbor a deformed TSC1/2 complex resulting from inactivating TSC2 phosphorylation at Ser664 by hyperactive MEK and ERK^60^. Given that DDIT4 requires the TSC1/2 complex to inhibit mTORC1^29^, these cells are essentially decoupled from YAP-mediated translational control.

Notably, we are also curious about the role of YAP during the expansion of the tumor mass. When primary tumors develop, the inner cells become progressively devoid of nutrients and oxygen due to their relative distance from neighboring vasculature^61^. As a result, these inner cells face not only nutrient deprivation but also hypoxia and consequently undergo necrosis^62^. As noted earlier, *DDIT4* is induced by hypoxia or serum starvation, and *Trail*, another YAP/TAZ-repressed target gene identified in our earlier study^6^, is a proapoptotic gene. On the basis of this logic, we hypothesize that tumors derived from YAP-active cells may be relatively more resistant to necrotic cell death given that *Trail* and *DDIT4* are suppressed (translation is sustained). Future studies aimed at investigating YAP tumor biology will be of great clinical interest given that whether necrosis promotes or impedes secondary cancer propagation still remains controversial.

## Supplementary information

Supplementary Information

Supplementary Table 1

Supplementary Table 2

Supplementary Table 3

## Data Availability

The Ribo-seq and RNA-seq raw data generated in this study have been deposited into the NCBI GEO database, series record GSE224264. All cell lines and plasmids are available upon request with a standard uniform biological materials transfer agreement (UBMTA).
