# Supplementary material for: YAP promotes global mRNA translation to fuel oncogenic growth despite starvation
Source: Exp Mol Med. 2024 Oct 1;56(10):2202–15. doi: 10.1038/s12276-024-01316-w (PMC11542038; doi:10.1038/s12276-024-01316-w)
Supplement: Supplementary file 1 — Supplementary Information [file 12276_2024_1316_MOESM1_ESM.pdf]

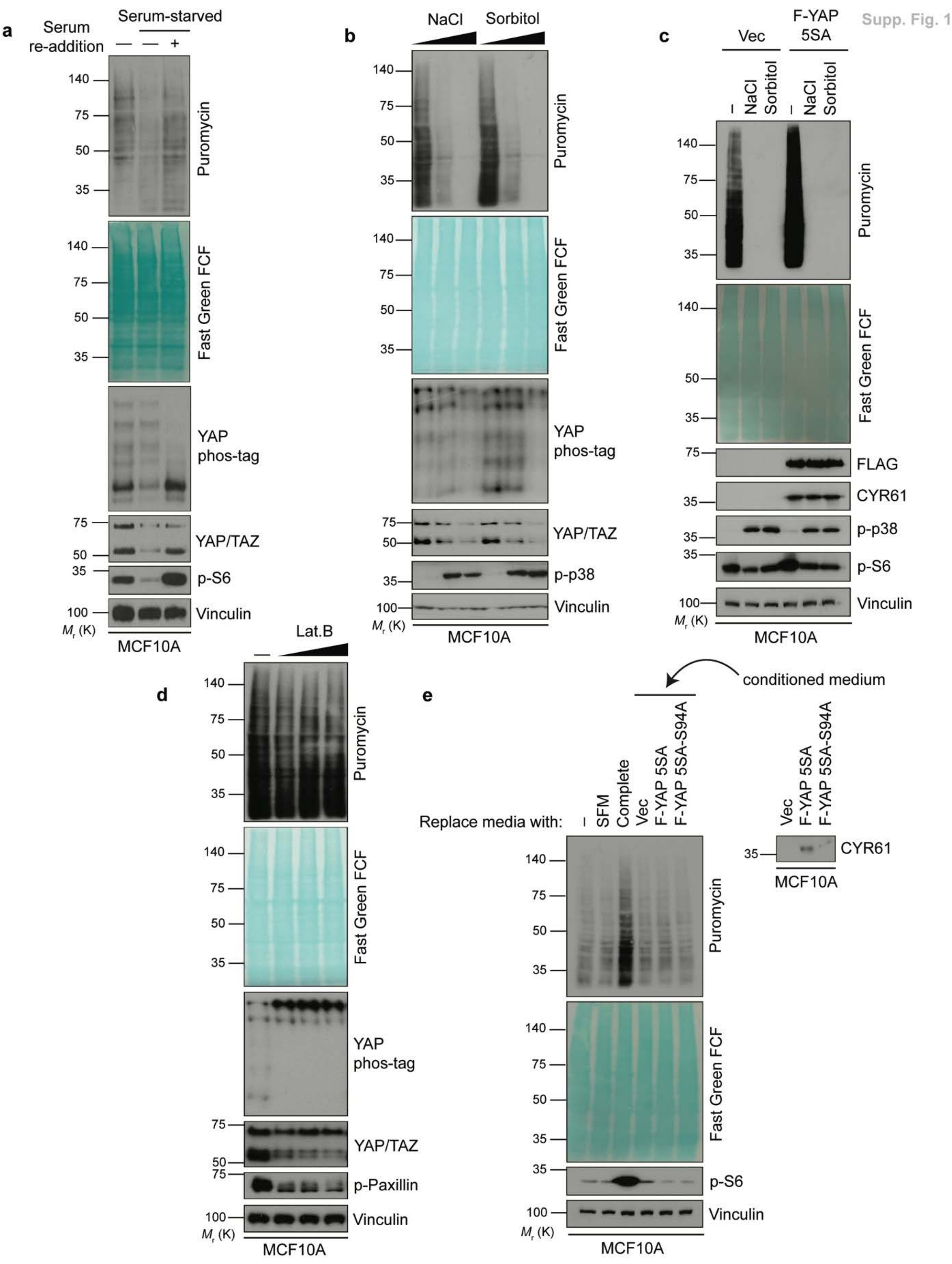
a
Serum re-addition
Serum-starved
— — +
140
75
50
35
Puromycin
Fast Green FCF
YAP phos-tag
YAP/TAZ
p-S6
100
Vinculin
M_r (K)
MCF10A
b
NaCl
Sorbitol
p-p38
c
Vec
F-YAP 5SA
– NaCl Sorbitol – NaCl Sorbitol
FLAG
CYR61
d
Lat.B
p-Paxillin
e
conditioned medium
Replace media with:
– SFM Complete Vec F-YAP 5SA F-YAP 5SA-S94A
Vec F-YAP 5SA F-YAP 5SA-S94A

Supplementary Figure 1 | Analysis of translation dynamics in cells exposed to extracellular stimuli that affect YAP/TAZ activity

a. MCF10A cells were starved (or not) of serum overnight, then incubated for 1 h with or without serum and then analyzed as in Figure 1A as well as YAP phosphorylation assessment using a Phos-tag gel.

b. MCF10A cells were treated with various concentrations of agents that induce osmotic stress — NaCl (50, 100, or 200 mM) or sorbitol (100, 200, 400 mM) — for 2 h and then analyzed as in Figure 1A as well as YAP phosphorylation assessment using a Phos-tag gel. Induction of phosphorylation of the mitogen-activated protein kinase p38 is indicative of activation of the MKK3-p38 stress response axis.

c. MCF10A cells stably expressing vector control or YAP 5SA were treated with NaCl (200 mM) or sorbitol (400 mM) for 2 h before analysis as in (A). YAP 5SA failed to reverse the inhibition of translation induced by osmotic stress. Note that S6 phosphorylation was not greatly affected by osmotic stress, suggesting that the effect of such stress on translation is largely independent of mTORC1.

d. MCF10A cells treated various concentrations of the actin-disrupting agent latrunculin B (Lat.B; 2.5, 5, or 10 μM) for 2 h were analyzed as in (A). Translation was not influenced by actin disruption despite efficient phosphorylation and inactivation of YAP. The decrease in the focal adhesion kinase (FAK)-mediated phosphorylation of paxillin indicates the efficient disruption of actin by latrunculin B.

e. Conditioned medium was collected from MCF10A cells stably expressing vector control, YAP 5SA, or YAP 5SA-S94A that had been starved of serum for 24 h, so as to exclude the

effects of serum and enrich for YAP-induced secreted factors. MCF10A cells that had been starved of serum overnight were then exposed for 1 h to conditioned medium, serum-free medium (SFM), or complete medium containing serum. The conditioned media were also subjected to immunoblot analysis for CYR61, showing that this secreted YAP target gene product was enriched in the conditioned medium from YAP 5SA-expressing cells.

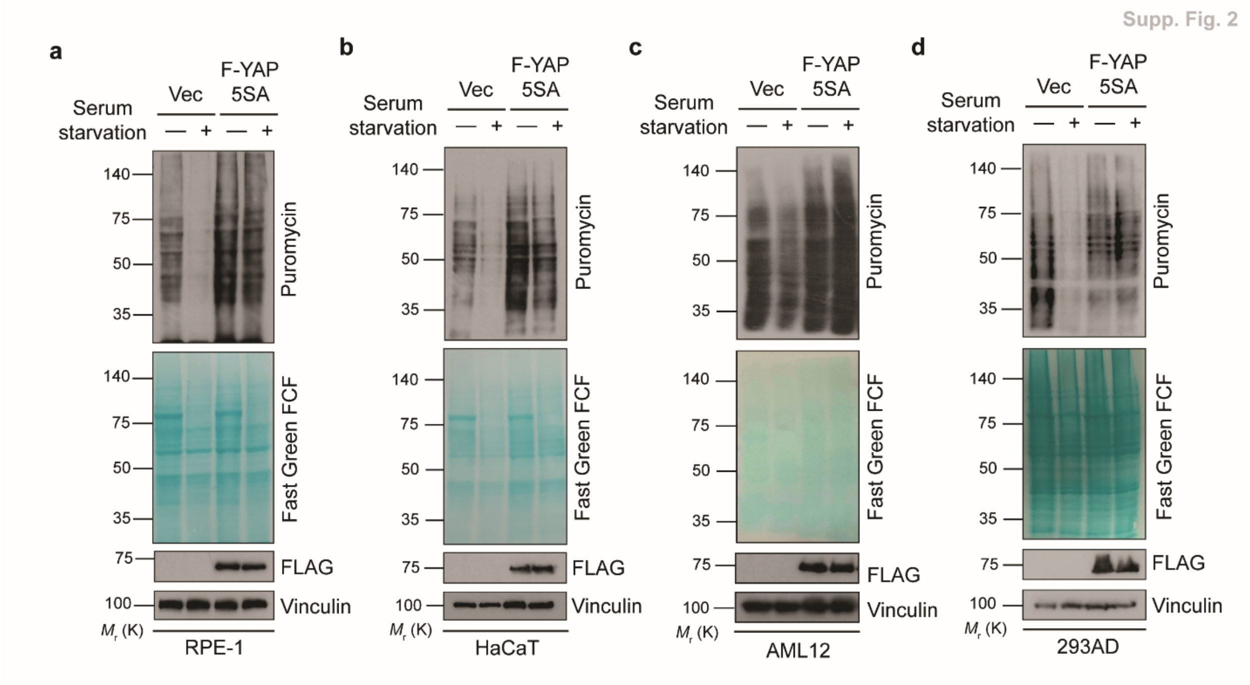

Supplementary Figure 2 | Efficient translation by YAP 5SA in the absence of serum is a generally conserved phenomenon

a-d. Relative translation of (a) retinal pigment epithelial cell line RPE-1, (b) human keratinocyte cell line HaCaT, (c) murine hepatocyte cell line AML12, or (d) human embryonic kidney cell line 293AD cells stably expressing vector control or YAP 5SA were starved (or not) of serum overnight and then analyzed as in Figure 1a.

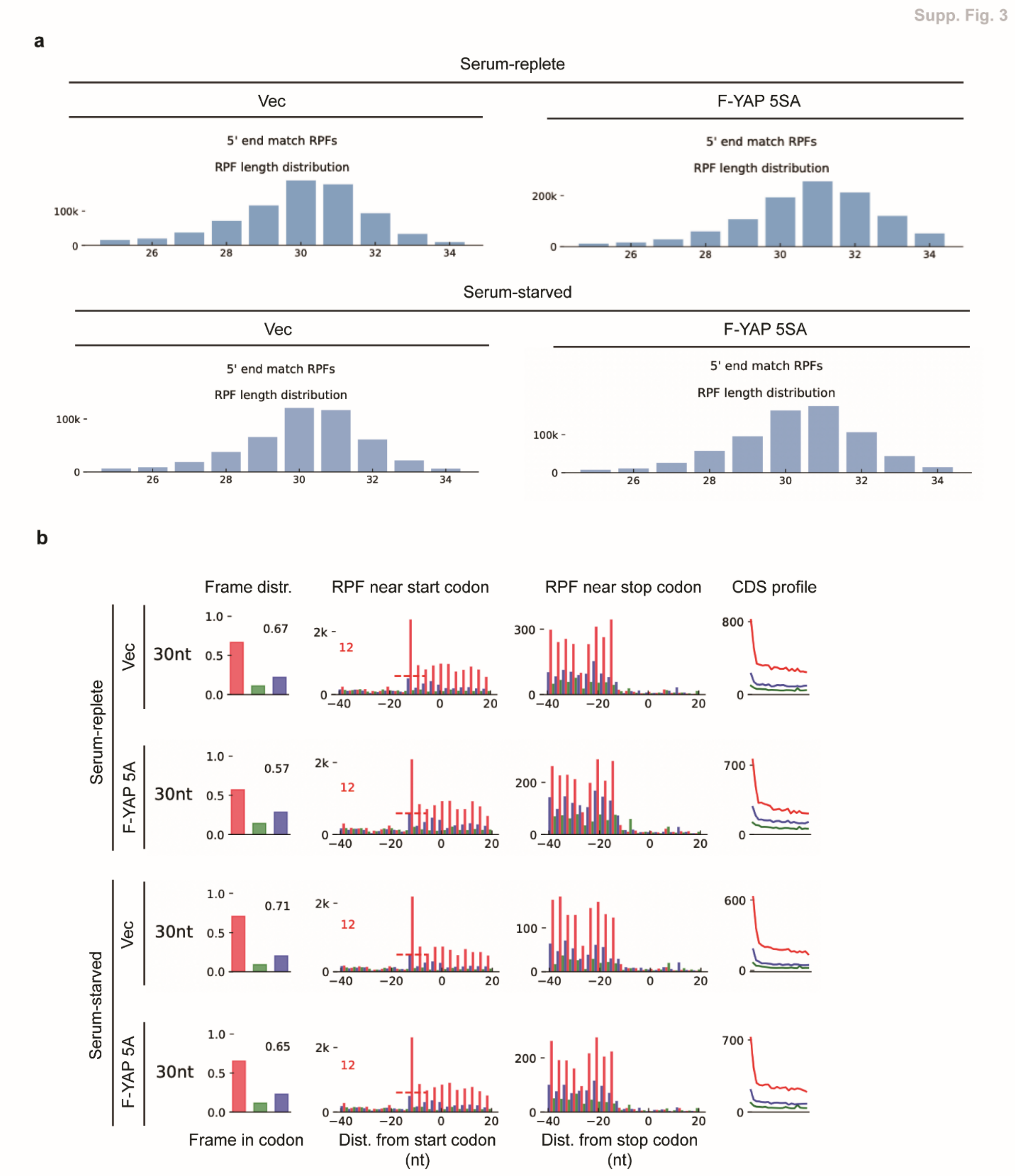

Supplementary Figure 3 | RPF length distribution and codon frame metrics of cells subjected to serum starvation and/or YAP 5SA overexpression

a. Quality control of Ribo-seq data with Ribo-TISH, comparing the length distribution of RPFs uniquely mapped to annotated protein-coding regions of indicated samples. Replicate #1 (of two) for each sample was selected for visualization.

b. Quality control of Ribo-seq data with Ribo-TISH, comparing quality profiles/metrics for RPFs of 30 nucleotide (nt) size mapped to protein-coding regions. Column 1: RPF distribution along the 5′ end across reading frames is shown. Column 2: RPF distribution along the 5′ end near annotated transcription initiation sites. Column 3: RPF distribution along the 5′ end near annotated stop codon. Column 4: RPF count profile across protein-coding regions across reading frames shown in Column 1. Replicate #1 (of two) for each sample was selected for visualization.

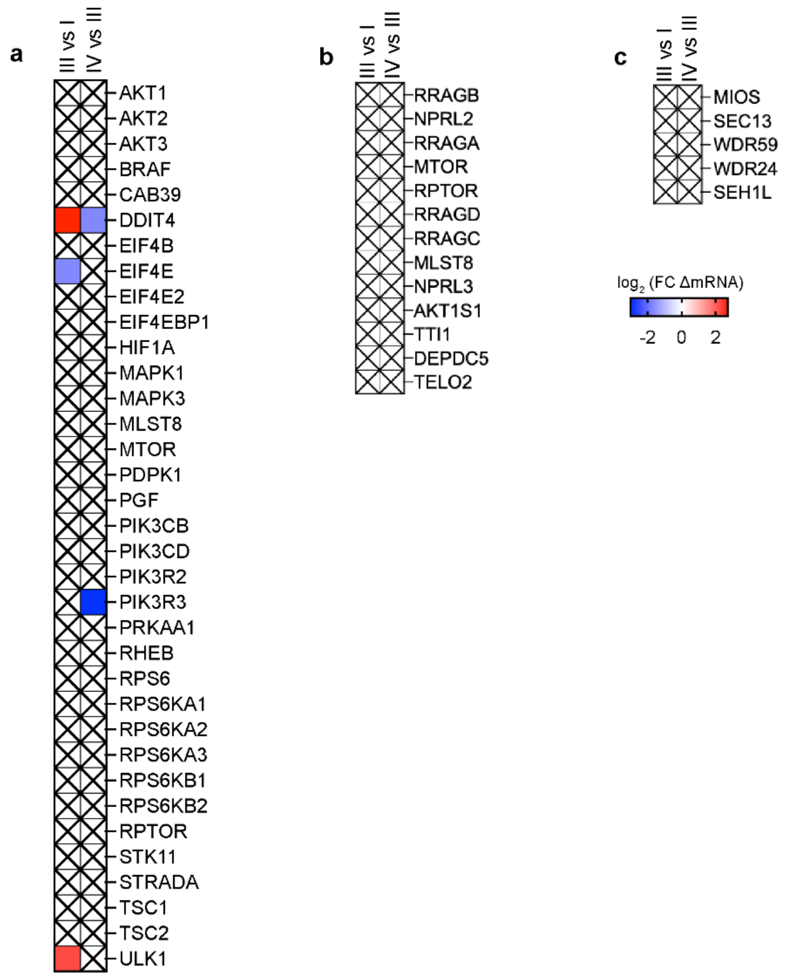

Supplementary Figure 4 | DEG analysis for mTOR pathway or related genes upon YAP 5SA overexpression or serum starvation

a. Heatmap illustration of pairwise differential gene expression for genes in Fig. 4a for components of the mTOR pathway ("KEGG_MTOR"). Crossed out ('X') boxes denote non-significant ($P_{adj} > 0.05$) comparisons, whereas significant ($P_{adj} < 0.05$) differentially-expressed genes with a $\log_2$(fold change) > 1.5 are colored according to the legend shown.

b. As in (a), for components of the GATOR1 complex ("KEGG_MEDICUS_REFERENCE_GATOR1_MTORC1_SIGNALING_PATHWAY").

c. As in (a), for components of the GATOR2 complex ("GOCC_GATOR2_COMPLEX").

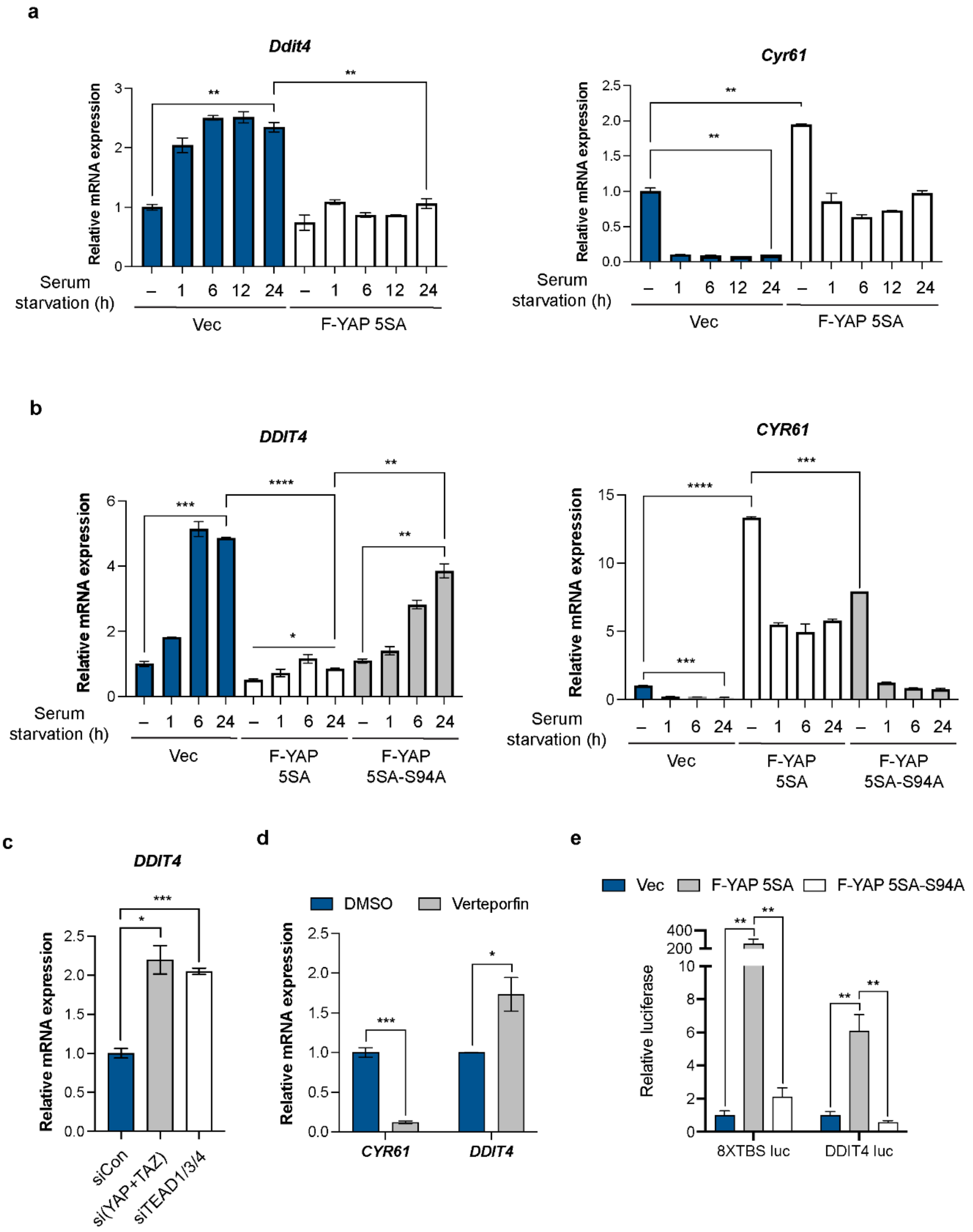

Supplementary Figure 5 | Transcriptional repression of *DDIT4* by YAP is dependent on TEAD

a. qRT-PCR analysis of *Ddit4* and *Cyr61* in AML12 cells stably expressing vector control or YAP 5SA starved of serum for the indicated times. Serum starvation-induced expression of *Ddit4* and its suppression by YAP 5SA were conserved in this mouse hepatocyte cell line. Data are means ± s.e.m. (n = 3 independent replicates). **P < 0.005 (unpaired Student's t-test).

b. qRT-PCR analysis of *DDIT4* and *CYR61* expression in MCF10A cells stably expressing vector control, YAP 5SA, or YAP 5SA-S94A, starved of serum for the indicated times. Data are means ± s.e.m. (n = 3 independent replicates). *P < 0.05, **P < 0.005, ***P < 0.0005, ****P < 0.0001 (unpaired Student's t-test).

c. qRT-PCR analysis of *DDIT4* expression in MCF10A cells transiently transfected with YAP/TAZ or TEAD1/3/4 siRNAs. Data are means ± s.e.m. (n = 3 independent replicates). *P < 0.05, ***P < 0.0005 (unpaired Student's t-test).

d. qRT-PCR analysis of *CYR61* and *DDIT4* expression in MCF10A cells treated with the YAP-TEAD inhibitor verteporfin (10 μM) for 24 h. Data are means ± s.e.m. (n = 3 independent replicates). *P < 0.05, ***P < 0.0005 (unpaired Student's t-test).

e. 293AD cells were transiently transfected with vector control or YAP 5SA or YAP 5SA-S94A constructs, along with a reporter plasmid containing either an 8X tandem TEAD-binding sequence (8XTBS) or DDIT4 promoter including the ORF (-1.5 kb ~ +1 kb relative to TSS). After addition of substrates to resulting cell lysates, firefly luciferase signals were

detected and normalized to that of Renilla luciferase. Data are means ± s.e.m. (n = 3 independent replicates). **$P < 0.005$ (unpaired Student's t-test).

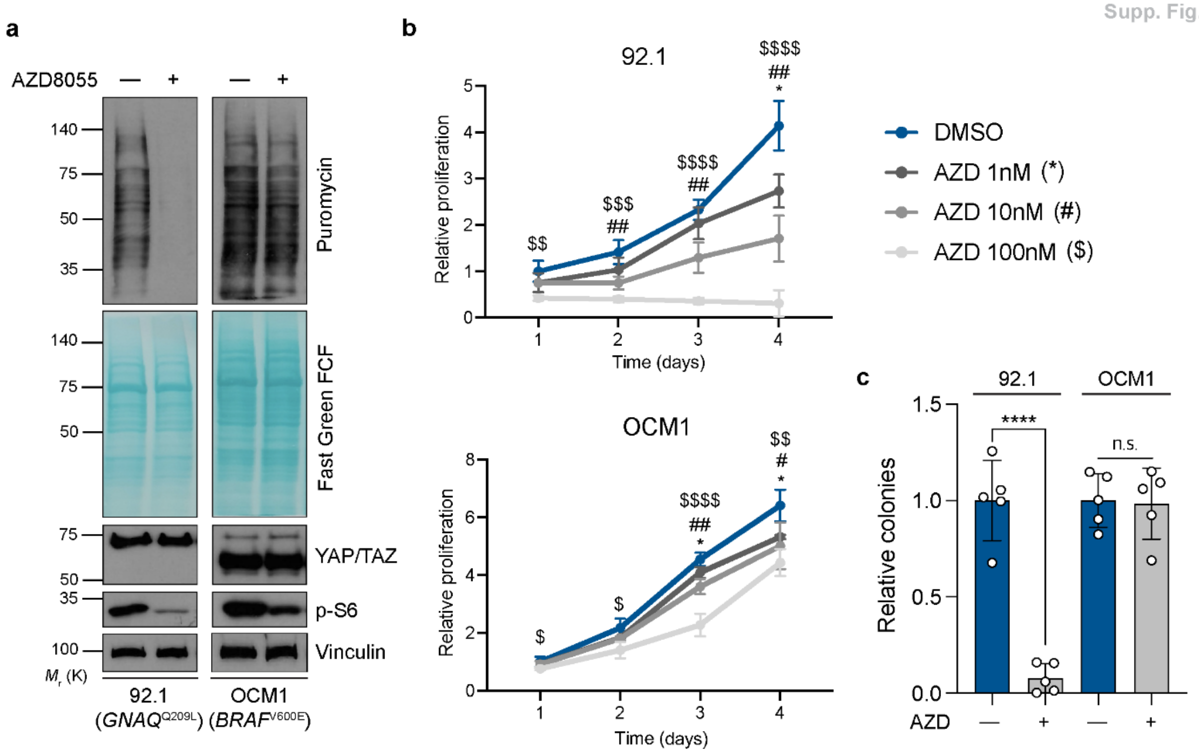

Supplementary Figure 6 | mTOR inhibitor treatment attenuates translation in and the tumorigenic potential of *GNAQ*-mutant, but not *BRAF*-mutant, uveal melanoma cells

a. 92.1 and OCM1 cells were treated with AZD8055 (100 nM) for 2 h and then analyzed as in Figure 1A.

b. Relative proliferation of 92.1 and OCM1 cells incubated in the presence of AZD8055 (1, 10, 100 nM) for the indicated times. Data are means ± s.e.m. (n = 4 independent replicates). The symbols *, #, and $ indicate comparisons between DMSO and AZD8055 at 1, 10, or

100 nM, respectively. */#/\$$P < 0.05$, ##/\$\$$P < 0.005$, \$\$\$$P < 0.0005$, \$\$\$\$$P < 0.0001$ (unpaired Student's *t*-test).

c. Relative anchorage-independent colony formation in soft agar by 92.1 or OCM1 cells incubated in the absence or presence of AZD8055 (100 nM) for 3 weeks. Data are means ± s.e.m. ($n = 5$ independent replicates). ****$P < 0.0001$; n.s., not significant (unpaired Student's *t*-test).

Supplementary Tables

Supplementary Table 1. Read count matrix of Ribo-seq samples

Supplementary Table 2. List of genes with differential translation efficiency (ΔTE) or mRNA expression (ΔmRNA)

Supplementary Table 3. List of genes inversely regulated by YAP 5SA and serum starvation
